# Author Correction: Evaluating FAIR maturity through a scalable, automated, community-governed framework

**DOI:** 10.1038/s41597-019-0248-6

**Published:** 2019-10-21

**Authors:** Mark D. Wilkinson, Michel Dumontier, Susanna-Assunta Sansone, Luiz Olavo Bonino da Silva Santos, Mario Prieto, Dominique Batista, Peter McQuilton, Tobias Kuhn, Philippe Rocca-Serra, Mercѐ Crosas, Erik Schultes

**Affiliations:** 1 Centro de Biotecnología y Genómica de Plantas, Universidad Politécnica de Madrid (UPM) – Instituto Nacional de Investigación y Tecnología Agraria y Alimentaria (INIA), Departamento de Biotecnología-Biología Vegetal, Escuela Técnica Superior de Ingeniería Agronómica, Alimentaria y de Biosistemas, Universidad Politécnica de Madrid (UPM), Madrid, Spain; 2 Institute of Data Science, Maastricht University, Maastricht, The Netherlands; 3 Oxford e-Research Centre, Department of Engineering Science, University of Oxford, Oxford, UK; 4 GO FAIR International Support and Coordination Office, Leiden, The Netherlands; 5 Leiden University Medical Center, Leiden, The Netherlands; 6 Institute for Quantitative Social Science, Harvard University, Cambridge, USA; 7 Leiden Center for Data Science, Leiden, The Netherlands; 8 VU Amsterdam, Amsterdam, The Netherlands

Correction to: *Scientific Data* 10.1038/s41597-019-0184-5, published online 20 September 2019

The affiliation of Mark Wilkinson and Mario Prieto was incompletely stated in the original publication. It is:

Centro de Biotecnología y Genómica de Plantas, Universidad Politécnica de Madrid (UPM) – Instituto Nacional de Investigación y Tecnología Agraria y Alimentaria (INIA), Departamento de Biotecnología-Biología Vegetal, Escuela Técnica Superior de Ingeniería Agronómica, Alimentaria y de Biosistemas, Universidad Politécnica de Madrid (UPM), Madrid, Spain

This has now been corrected in the HTML and PDF versions.

